# Supplementary material for: Exploring Elementary Students’ Social-Emotional Development Through Dialogic Pedagogy: Insights from Cinematic Narratives
Source: Behav Sci (Basel). 2025 Dec 8;15(12):1701. doi: 10.3390/bs15121701 (PMC12729373; doi:10.3390/bs15121701)
Supplement: Supplementary file 1 [file behavsci-15-01701-s001.zip › behavsci-3908225-supplementary.pdf]

Supplementary document 1. Steps of the Analysis Process

1. **Data Collection and Transcription**
   a. The interviews were analysed verbatim.
   b. Data was written in Word.
2. **Independent Open Coding**
   a. Two researchers (Researcher A and Researcher B) independently coded the data.
   b. More than one code was allowed for each semantic unit.
3. **Code Comparison and Compliance Analysis**
   a. The researchers compared their codes
   b. Coefficient of concordance = .79 (high level of concordance).
4. **Creating the Code Book**
   a. Code definitions, sample quotes, and scope are clearly written
   b. Categories were created based on the relationship between the codes.
5. **Converting Categories to Themes**
   a. Similar categories were grouped together according to their semantic integrity.
   b. The themes were clarified after discussions between researchers.
6. **Member Checking and Researcher Triangulation**
   a. The findings were presented to the teachers and their accuracy was checked.
   b. Researchers made comparative interpretation throughout the process.
7. **Reporting Themes**
   a. Sub-themes and representative child expressions were reported under each theme.
   b. The findings were detailed with “thick description.”

Supplementary document 2. Code Book

Theme 1: Social-Emotional Awareness

Category 1.1: Self-Awareness

K1.1.1 – Describing Emotion (code): The child names his or her own emotions or states that he or she is aware of them (description)

K1.1.2 – Expressing Strengths/Weaknesses: Indicating their own competencies or areas of difficulty

Category 1.2: Emotional Regulation

K1.2.1 – Emotional Control Strategy: The regulation methods used in situations such as anger, excitement, sadness, etc.

K1.2.2 – Support Search: Indication of help from teachers, family, or friends

Theme 2: Social Relations and Empathy

Category 2.1: Empathic Response

K2.1.1 – Understanding Others' Emotions: Saying that he understands what his/her friend feels

K2.1.2 – Support/Shoutout: Attempting to help after empathy

Category 2.2: Social Skills

K2.2.1 – Partnership: Teamwork or cooperative behaviour

K2.2.2 – Conflict Resolution: Strategy for resolving disagreements with friends

Theme 3: School Environment and Teacher Support

Category 3.1: Relationship with the Teacher

K3.1.1 – Sense of Trust Stating that he/she communicates comfortably with the teacher

K3.1.2 – Perception of Support   Stating that the teacher listens to him/her and supports him/her

Category 3.2: Classroom Environment

K3.2.1 – Safe Environment: Stating that the classroom is a safe, respectful place

K3.2.2 – Peer Relationship: Positive/negative statements about classmates

Theme 4: Attitudes Towards Social-Emotional Learning Activities

Category 4.1: Likes and Interests

K4.1.1 – Liking the Event: Positive evaluation of the implemented SEL activity

K4.1.2 – Perception of Learning: Stating that the activity contributed to him/her

Category 4.2: Challenges / Criticisms

K4.2.1 – Difficulty in Comprehension: Indicating that the activity was difficult, complex, or incomprehensible

K4.2.2 – Participation Barrier: Situations that reduce participation such as shame, shyness, time problems, etc.
